# Caries arresting effect of silver diamine fluoride on dentine carious lesion with S. mutans and L. acidophilus dual-species cariogenic biofilm

**DOI:** 10.4317/medoral.18831

**Published:** 2013-05-31

**Authors:** May L. Mei, Chun H. Chu, Kan H. Low, Ching M. Che, Edward CM. Lo

**Affiliations:** 1 Faculty of Dentistry, The University of Hong Kong, Hong Kong, China; 2 Affiliation of State Key Laboratory of Synthetic Chemistry, Department of Chemistry, The University of Hong Kong, Hong Kong, China

## Abstract

Objectives: This in vitro study investigated the effects of silver diamine fluoride (SDF) on dentine carious lesion with cariogenic biofilm. Study Design: Thirty human dentine blocks were inoculated with Streptococcus mutans and Lactobacillus acidophilus dual-species biofilm to create carious lesion. They were equally divided into test and control group to receive topical application of SDF and water. After incubation anaerobically using micro-well plate at 37oC for 7 days, the biofilms were evaluated for kinetics, morphology and viability by colony forming units (CFU), scanning electron microscopy (SEM), and confocal microscopy (CLSM), respectively. The carious lesion underwent crystal characteristics analysis, evaluation of the changes in chemical structure and density of collagen fibrils using x-ray diffraction (XRD), Fourier transform infrared spectroscopy (FTIR) and immune-labeling. Results: The log CFU of S. mutans and L. acidophilus in the test group was significantly lower than control group. SEM and CLSM showed confluent biofilm in control group, but not in test group. XRD showed the loss of crystallinity of dentine due to the dissolution of hydroxyapatite crystal structure in test group was less than control group. FTIR showed that log [Amide I: HPO42-] for test vs. control group was 0.31±0.10 vs. 0.57±0.13 (p<0.05). The gold-labeling density in test vs. control group was 8.54±2.44/µm2 vs. 12.91±4.24/µm2 (p=0.04). Conclusions: SDF had antimicrobial activity against the cariogenic biofilms and reduced demineralization of dentine.

** Key words:**Caries, caries arrest, dentine, silver, silver diamine fluoride, fluoride, biofilm,cariogenic.

## Introduction

A recent review on silver diamine fluoride (SDF) concluded that SDF is a safe, effective, efficient, and “equitable” caries control agent that can be used to help meet the World Health Organization Millennium Goals and fulfill the US Institute of Medicine’s criteria for 21st century medical care (1). Milgrom and Chi (2) advocated that SDF therapy is an important prevention-centered caries management strategy. Recent studies suggested that topical application of SDF is a simple, cost-effective and noninvasive method in caries management (3). Clinical trials showed that SDF prevented and arrested coronal caries in primary teeth in preschool children (4) and in permanent teeth in older children (5). Dos Santos Jr reported that SDF is a better option to control caries than intermediate restorative treatment with glass ionomer in primary teeth (6). Laboratory studies found SDF could prevent *Streptococcus mutans* biofilm growth on demineralized and non-demineralized dentine (7). SDF was also demonstrated possessing an anti-microbial activity against cariogenic mono-species biofilm of *S. mutans* or Actinomyces naeslundii formed on dentine surfaces (8). These studies, however, lack interactions between bacteria in a multi-species biofilm at real caries situation. Characterization of the microbial interaction is necessary to understand pathogenesis of dental caries (9).

*L. acidophilus* were frequently found in high numbers in both superficial and deep carious lesions (10). Lactobacilli cannot form plaque on the tooth on their own and depend on extracellular polysaccharide produced by other oral organisms, mainly streptococci, for colonization. The capacity of Lactobacilli to form biofilm was enhanced significantly in the presence of *S. mutans* (11). *S. mutans* and *L. acidophilus* produce lactic acid from fermentable sugar and are able to live in highly acidic environments. *S. mutans* is important for the initiation and progression of caries (12). Fermented acid produces an acidic environment which is favorable for Lactobacilli to grow. *S. mutans* and *L. acidophilus* are often considered the two most important cariogenic bacteria associated with dentine caries (13). However, no publication in English so far reported the mechanism of SDF effect on dual-species biofilm.

The methodology used in previous teeth hard tissue studies mostly focused on the mechanical or radiographical changes of caries lesion, such as microhardness testing, microradiography, Arends et al. (14). The results mainly reflect the changes in mineral content of caries lesion. However, changes of caries in microstructure are usually associated with variation in both mineral and organic content (15). Electrochemical methods are solid methods to detect any changes in mineral constituents which reflect the microstructure of hard tissue (15). Immunohistochemistry labeling could detect both the amount and location of collagen fibers (16). Therefore, electrochemical (include x-ray diffraction (XRD), Fourier transform infrared spectroscopy (FTIR)) and immunochemical (immune-labeling) methods were used in this study. This strategy can provide comprehensive and integral insight into microstructural changes of dentine caries under biofilm challenge. This is also the first study which investigates SDF effect on dentine caries in consideration of both dual-species biofilm and dentine structure changes.

Therefore, this in vitro study aimed to investigate the anti-microbial effect of SDF on *S. mutans* and *L. acidophilus* co-cultured dua-lspecies biofilm as well as dentine caries lesions. The null hypothesis of the study was that SDF has no anti-microbial effect on the dual-species biofilm formed on dentine carious lesions.

## Material and Methods

-Sample preparation

**Figure 1:**
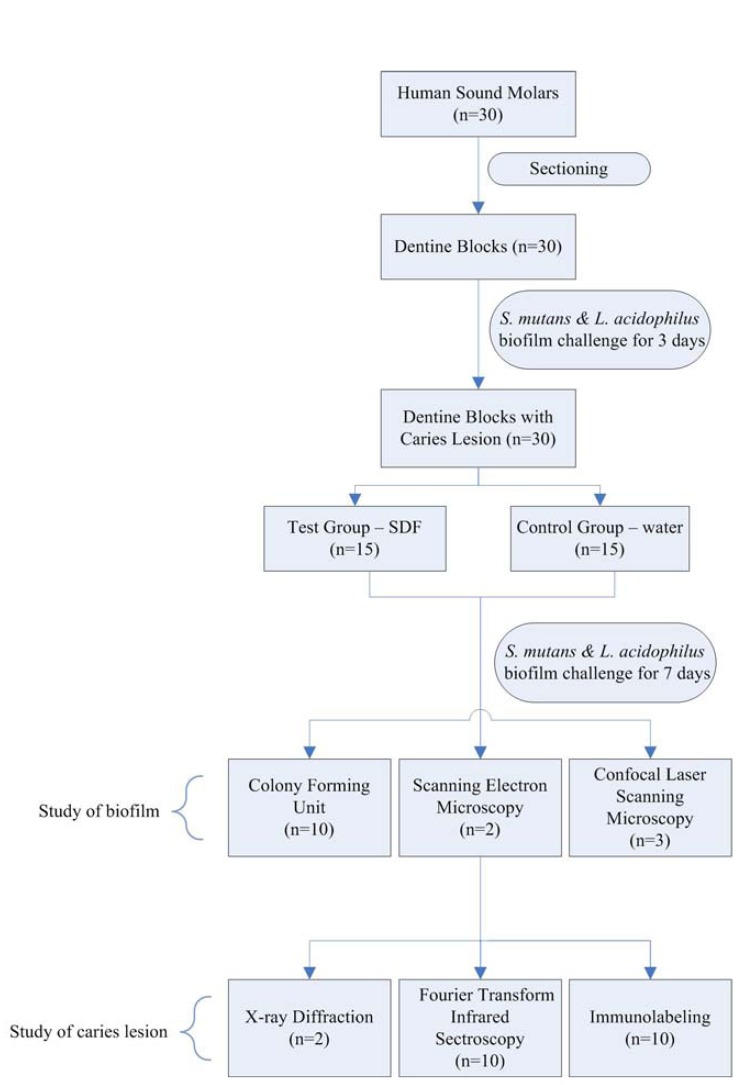
Flow chart of experiment process.

This study was approved by a local Institutional Review Board (IRB UW08-052). Extracted sound human third molars were collected with written consent from patients. The teeth were stored in 1% sodium azide at 4oC. Thirty dentine blocks of 2×2×4 mm3 were prepared from the sound human molars. The dentine blocks were examined under a stereomicroscope (×10 magnifications) to ensure they had no cracks, hypoplasia, or white spot lesions. The surfaces of dentine blocks were polished by micro-fine 1,200 sanding paper under water using Ecomet® 6 grinder- polisher (Buehlar, Waukegan, USA). The dentine blocks were treated with 1% citric acid for 5 min to eliminate the smear layer on the surfaces, and they were then rinsed with distilled water. Half of the surface of each block was coated with an acid-resistant nail varnish (Clarins, Paris, France) to serve as an internal control. The blocks were then sterilized with ethylene oxide (Amsco Eagle 2017 EO sterilizer; STERIS, Mentor, USA) for 16 hours (17). Figure 1 showed the flowchart of experimental process.

*S. mutans* American Type Culture Collection (ATCC) 35668 and*L. acidophilus* ATCC 9224 were cultured on blood agar plates at 37oC for 2 days anaerobically. A single colony was picked from each plate to prepare 24-hour broth cultures in basal medium supplemented with 5% glucose (BMG medium) at 37oC under anaerobic conditions. Subsequently, bacterial cell pellets were harvested and resuspended in BMG to a cell density of McFarland 3 (109 cells/mL). A 300µL aliquot of each bacteria culture was mixed and inoculated on each dentine block sitting in a well of a 24-well plate with BMG. The plate was placed in an anaerobic chamber at 37oC for 3 days. The medium was refreshed daily without disturbing dentine surface. This generated dentine lesion about 80 µm in depth.

Subsequently, fifteen dentine blocks were underwent topical application of a commercially available 38% SDF solution (Saforide; Toyo Seiyaku Kasei Co. Ltd., Osaka, Japan) on exposed surfaces with a gravimetric micro-brush according to manufacturer´s instruction. The mean (±SD) amount of SDF applied was 0.22 mg ± 0.07 mg (or 8.8 µg ± 2.8 µg fluoride), as estimated by calculating the difference of the micro-brush before and after application. The other 15 blocks were treated with distilled water as a control. The SDF was applied within 5 seconds for each block. After treatment, all the dentine blocks were returned to the 24-well plate immediately with BMG and placed on an incubator-shaker (Incubator-shaker 3525; Labline, Mumbai, India) set at 75 rpm inside the anaerobic chamber for 7 days at 37oC. Then dentine blocks with biofilms were taken out of the well-plate for further assessment.

-Study of biofilms 

-CFU count and pH measurement

Growth kinetics of the dual-species biofilm was assesssed by determining bacterial counts in colony-forming units (CFU). Selective media agar plates of Mitis Salicarius, Rogosa were used for *S. mutans* and *L. acidophilus* respectively. As fluoride ions might react with SiO2 and cause inaccurate pH reading by a pH sensor, this study used pH test paper (Macherey-nagel, Düren, Germany) to measure the resting pH of the biofilm. The pH paper showed pH value at 0.5 interval from 4.5 to 7.5 (eight intervals).

-Confocal laser scanning microscopy

Confocal laser scanning microscopy (CLSM) was used to study the viability of bacteria in biofilms on dentine carious lesions. Biofilms were labeled in situ using two fluorescent probes: PI and SYTO-9 (LIVE/DEAD BacLight Bacterial viability kit; Molecular Probes, Eugene, OR, USA). The red PI probe labels dead cells whereas the green SYTO-9 probe labels live cells. Dentine blocks were incubated in the dark for 30 min after labeling (18). Thereafter, 5 cellular images of eachbiofilm specimen were obtained using CLSM (Fluoview FV 1000, Olympus, Tokyo, Japan) and examined using special image analysis software (Image J; National Institutes of Health, USA). The red-to-green ratio was calculated to indicate the ratio of dead-to-live bacteria on the anti-microbial effect of SDF.

-Scanning electron microscopy 

Scanning electron microscopy (SEM) was used to examine the topographical features of the biofilm. In preparation for SEM, dentine blocks with biofilm were rinsed in 4% (vol/vol) formaldehyde followed by 1% (vol/vol) PBS; they were then placed in 1% osmium tetroxide solution for 60 min. Then, they were rinsed with distilled water and dehydrated in a series of ethanol solutions at increasing concentrations (70% for 10 min, 95% for 10 min, and 100% for 20 min). Dentine blocks were then dried in a desiccator and sputter-coated with gold. The surface topographies of biofilms were studied under SEM (Leo 1530, LEO, Ober-kochen, Germany) at 12 kV in high-vacuum mode.

-Study of caries lesion

After biofilm collection, the underneath dentine block were used for XRD to analyze crystal characteristics and FTIR testing to evaluate any change in chemical structure. The surface of the caries lesion was also used for immunochemistry study to evaluate the quantity of intact collagen fibrils.

-X-ray diffraction analysis

Step-scanned lock-coupled XRD data was collected by BRUKER D8 ADVANCE X-ray powder diffractometer with CuKa (l = 1.5418 Å) radiation equipped with a scintillation counting detector. The accelerating voltage and the applied current of the X-ray generator were 40 kV and 40 mA, respectively. The X-ray beam was paralleled via a Göbel mirror, and confined by a divergence limiting slit (0.6 mm) and a Soller slit before reaching the sample to reduce axial divergence of the incident beam. A receiving slit (0.6 mm) and a detector slit (0.2 mm) were employed to increase resolution. Data collection parameters: 2q range = 20–60o, step size = 0.05o, scan speed = 30 second/step, the positions of diffraction lines (002), (112) and (300) were determined by the peak top method. After a preliminary data collection, the diffraction data was recollected to minimize systematic errors. Reproducible dataset of each sample was obtained and the sample damage by X-ray irradiation was negligible. The phase purity and indexing of the hydroxyapatite (HAP, Ca5 (PO4)3OH) phase was checked by International Center for Diffraction Data (ICDD, PDF-2 Release 2004) database match search. The diffraction patterns were analyzed with BURKER DIFFRAC plus EVA program.

-FTIR analysis

The analysis of potential changes in the chemical structure of dentine lesions was performed using FTIR spectroscopy with a Bio-Rad FTIR UMA 500 machine (Bio-Rad Laboratories, Hercules, California, USA). The chemical structure was calculated from the spectrally derived matrix-to-mineral ratio (the ratio of the integrated area of protein amide I absorbance from 1585 to 1720 cm-1 to that of phosphate [HPO42-] absorbance from 900 to 1200 cm-1), Spectra for demineralized dentine lesions (n=10 for each bacteria group) were obtained by the average acquisition of data at the spatial resolution achieved with a 100×100 µm2 aperture over the lesion surface. The log value of the [amide I: HPO42-] absorbance ratio was then used as an indicator of the extent of demineralisation of dentine due to the carious activity of the biofilm (8,19).

-Immunochemistry

The type I collagen in lesion surfaces were labeled by immunolabeling method proposed by Breschi (16). The dentine blocks were put in an ultrasonic bath with de-ionized water (pH 7.4) for 5 min in and exposed to 10% citric acid for 15 sec. Then the blocks were rinsed in a 0.05M TBS at pH 7.6 for 15 min. Antigen saturation was obtained with normal goat serum (NGS, BioCell International, Cardiff, UK) in TBS (0.05 M at pH 7.6) for 30 min at 23oC. Incubation was performed at 4oC using primary antibody: a mouse IgG anti-Type I collagen (Sigma Chemical Co., MO, US). Gold labeling was performed using secondary antibody: a goat IgG anti-mouse IgG (Electron Microscopy Science, PA, US) conjugated with 25 nm of colloidal gold particles for the Type I collagen. Secondary antibody were diluted in 0.02 M TBS at pH 8.2 and applied at room temperature. After the incubation, the dentine blocks were rinsed in distilled water and fixed in 2.5% glutaraldehyde. After dehydration in an ascending percentage of 70%, 80% 90% and finally 100% ethanol solution, the specimens were hexamethyldisilazane-dried. The specimens were prepared and examined under SEM (Leo 1530, Oberkochen, Germany) at 20 kV. Micrographs with the same magnification were obtained for each sample (n=10 in a group). The labeling index was calculated as the mean of the gold particle number/µm2 (±SD) of visible organic network obtained from images from each sample. The SEM images of each group were taken. The labeling index for collagen fibrils was determined.

-Statistical analyses

Parametric t test was used to compare log CFU, log [amide I: HPO42-] ratio, and number of gold particles between SDF-treated and control groups at the same lesion depth. All analyses were conducted using IBM SPSS Statistics 19.0 Statistics (IBM, Armonk, NY, USA). The cutoff level of significance was taken as 5% for all analyses.

## Results

-Biofilm characteristics

As  Table 1 showed, log CFU counts of both *L. acidophilus* and *S. mutans* in SDF group were significantly lower than that in control groups (p<0.01). The dead-to-live ratios from CLSM images, which indicate strength of anti-microbial effect, were significantly higher after topical SDF application than after water application (p=0.03,  Table 1). The pH value of control groups was between 3.5-4.0, and the value increased to 6.0-6.5 with SDF treatment. These findings were also corroborated by the results with SEM and CLSM in the following sections.

**Table 1:**
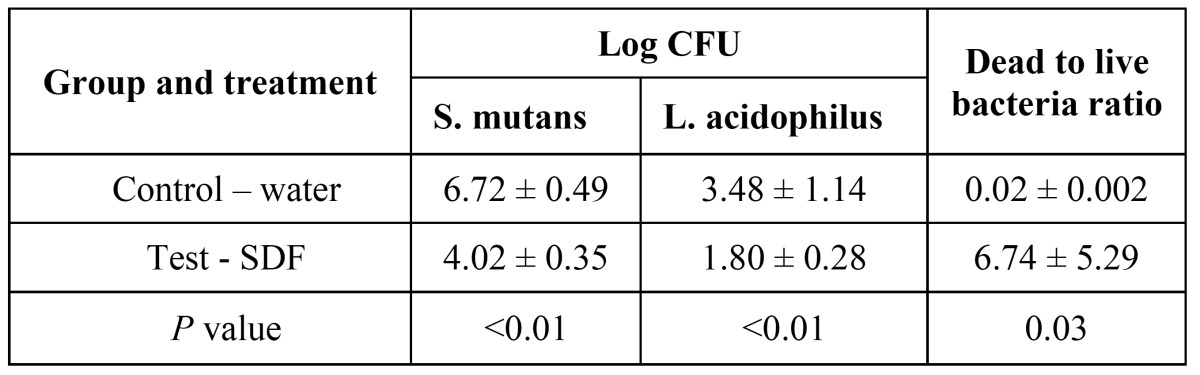
Bacterial counts (log CFU), ratio of dead-to-live bacteria of dual-species biofilm (n=10).

**Figure 2:**
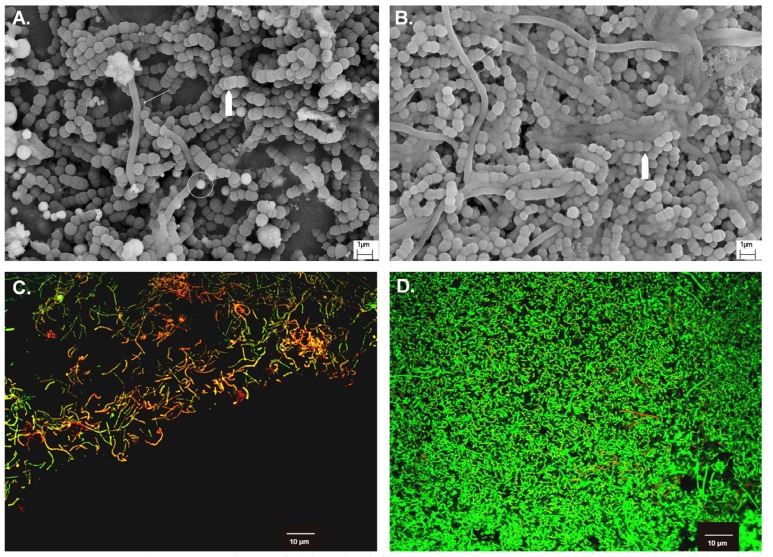
SEM (×15,000) and CLSM (×600) images of dual-species biofilm. SEM: A. Test - SDF; B. Control – water; Arrow: L. acidophilus; Pentagon: S. mutans; Circle: silver particle. CLSM: C. Test - SDF; D. Control – water; Live bacterial cells appeared green whereas, dead cells as red.

In SDF treatment groups, round particles of about 0.5 to 1µm that visible in SEM images (Fig. 2) were confirmed to be silver by Energy Dispersive x-ray Spectrometry. In CLSM images, a majority of bacteria present in the biofilm fluoresced red in SDF treatment group (Fig. 2), indicating that the bacteria were mostly dead after SDF application. In control groups, SEM images showed L. acidophilus formed long rod structure intertwined with *S. mutans* (Fig. 2). CLSM images of biofilms demonstrated most areas of the biofilms were predominant green (alive bacteria) and few dead cells (appeared red) were seen among live bacterial cells (Fig. 2).

-Hard tissue characteristics

**Figure 3:**
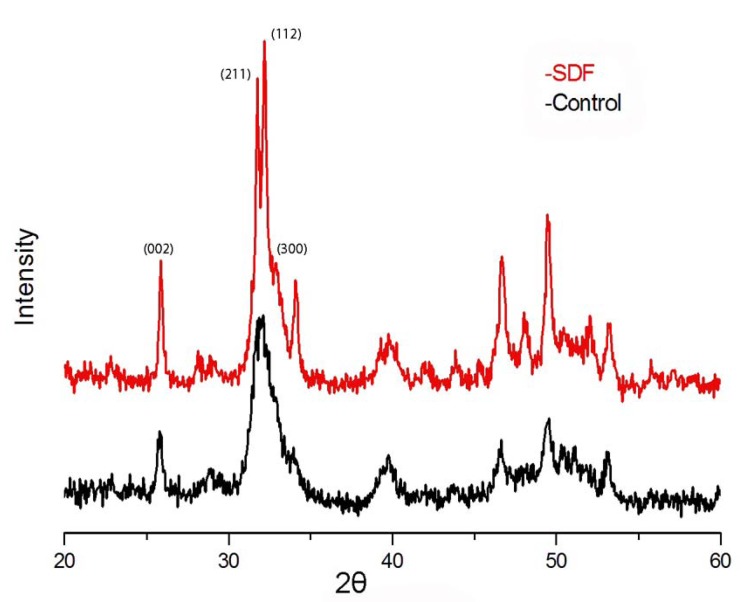
Typical XRD patterns on SDF and control samples.

Typical XRD spectra of the SDF and control groups are shown in figure 3. The XRD analysis indicated that the main crystal composition on the surface of dentine blocks in the control group was corresponding to HAP crystallized in Hexagonal P63/m with a = b = 9.432 Å, c = 6.881 Å, a = b = 90o, g = 120o. In control group that treated with water, the diffraction peaks of Miller’s indices (Miller’s indices are a symbolic vector representation for the orientation of an atomic plane in a crystal lattice and are defined as the reciprocals of the fractional intercepts which the plane makes with the crystallographic axes) at (002) and (211) broadened when compare with SDF group.

The values of log [amide I: HPO42-] are showed in  Table 2. The log [amide I: HPO42-] ratio was lower in SDF group than that in the control (p=0.01). The mean gold particles densities in SDF group and control group were 8.37±2.28/µm2 and 6.45±2.12/µm2, respectively ( Table 2).

**Table 2:**
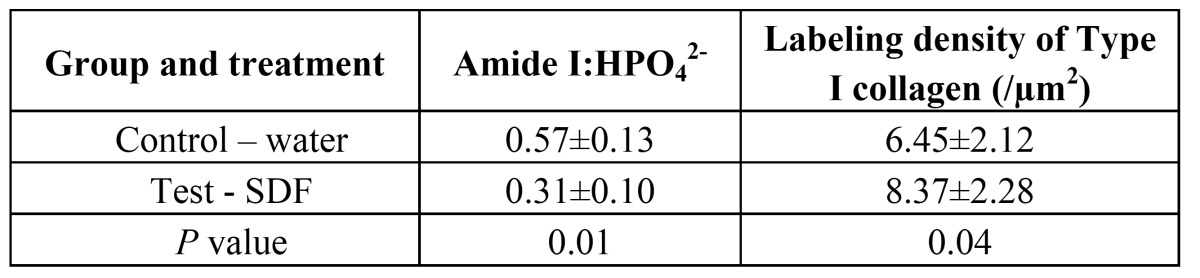
Log FTIR [amide I: HPO42-] and labeling density of Type I collagen of lesion surfaces and treatment groups (n=10).

## Discussion

This study provides essential information on the anti-cariogenic effects of SDF and the cariogenic effects of a dual-species biofilm on dentine. *S. mutans* and *L. acidophilus* are considered two most important bacteria associated with dentine caries (13). This dual-species cariogenic biofilm is relatively stable and easy to handle in this in vitro study. When compare to mono-species biofilm, dual-species include the interaction between two species and provide a means for studying a more complex microbial ecosystems. However, it is different from the in vivo multi-species plaque biofilms in both survival and pathogenic potential. Therefore, the results cannot be extrapolated to the in vivo situation and caution should be exercised in their interpretation.

SDF is available in various concentrations, such as 38%, 30% and 12%. This study used 38% SDF because it is the most commonly used concentration (20). When comparing with previous study which has showed SDF has antimicrobial effect on mono-species of *S. mutans* (8), increased antimicrobial tolerance of the species in dual-species was observed in current study. The values of CFU countings in mono-species biofilm treated by SDF were nearly zero (8), but the values were increased in this study. Under a more complex environment (dual-species biofilm), bacteria seems to survive better when compare with mono-species. The results corroborated with the findings by Kara et al. which showed dual-species biofilms of two oral bacteria (*S. mutans* and *Veillonella parvula*) were less susceptible to chlorhexidine when compared with mono-species biofilm (21). Cowan et al. (22) also reported commensal relationships in dual-species biofilms caused adaptive strategies and increased survival. The mechanisms of increased antimicrobial tolerance in dual-species biofilms are not clear so far, but it was suggested that cell to cell communication may play a role (23). The presence of the second species may alter the composition and viscosity of the extracellular polysaccharide matrix and may slow down the penetration of antimicrobial into the biofilm (24). Physiological changes may occur when two species are able to transfer conjugative plasmids and thus share protective mechanisms (23) or support each other by complementing enzymes that are necessary to manage environmental challenges (25).

XRD is a valuable method to identify crystal structure. In this study, it was used to characterize the changes in HAP crystal structure on the surface of dentine blocks. Figure 3 showed that (002), (211) and (300) lines of SDF-treated samples are well separated, on the contrary, those of the control sample are poorly defined, which indicates that the crystallinity of HAP in SDF group is higher than that in control. Moreover, the diffraction peaks of Miller’s indices of control group at (002) and (211) were broadened when compare with SDF group, indicating the loss of crystallinity of dentine due to the dissolution of HAP crystal structure of by the acid ions produced by the bacterial mediated reaction (15).

Presumably, acids from the oral biofilm dissolve HAP and expose the previously HAP-masked collagens and organic matrices, thereby generating more carbonyl groups (26). Amide I band at 1691 cm-1 was applied as the organic content of the tooth (from breakdown of type I collagen) while HPO42- was used as the mineral density. The log [amideI: HPO42-] absorbance value was applied as an indicator of the extent of demineralization of tooth tissue due to the carious activity of the oral biofilm. Larger log values corresponded to a greater extent of demineralization (8). Therefore, less extent of demineralization of dentine in SDF group when compare to control group. A laboratory study reported that SDF can inhibit Matrix metalloproteinases (MMPs), this also contribute the inhibition of collagen degradation in dentine (27).

FTIR could be a reflection of extent of demineralization. However, the values obtained are relative values and cannot predict the exact changes between different groups. Dentine contains about 20 wt% or 30 vol% organic component. Approximately 90% of the organic phase consists of type I collagen (28). Type I collagen fibrils provide a three-dimensional scaffold for the deposition of the apatite mineral phase (29). The antibody anti-helical portion used in immunochemistry recognizes the substrate based on three-dimensional conformation that is related to the presence of an intact triple helix of collagen I (30). Since the antibody recognizes the native form of collagen type I and does not react with the denatured molecule, the number of the gold particles could represent the amount of sound collagen I in the dentine surface.  Table 2 showed that the mean labeling density for Type I collagen in SDF group was higher than that in control, which indicates there are more sound collagen I in the test group than control group. These results are consistent with XRD and FTIR study. Less collagen I were exposed and destroyed in the test group and SDF protect the dentine surface from further demineralization. SDF was applied on the biofilm in current study and ceased the caries progression, the 38% SDF’s could have a significant antimicrobial effect on the dual species cariogenic biofilm.

The present study comprehensively showed that SDF possess an anti-microbial activity against *S. mutans* and *L. acidophilus* dual-species cariogenic biofilms formed on dentine surfaces. In addition, SDF slowed down demineralization of dentine and protect the collagen from being destroyed. This dual activity could be the reason behind clinical success of SDF.
